# Structural Insights on Retroviral DNA Integration: Learning from Foamy Viruses

**DOI:** 10.3390/v11090770

**Published:** 2019-08-22

**Authors:** Ga-Eun Lee, Eric Mauro, Vincent Parissi, Cha-Gyun Shin, Paul Lesbats

**Affiliations:** 1 Department of Systems Biotechnology, Chung-Ang University, Anseong 17546, Korea; 2 Fundamental Microbiology and Pathogenicity Laboratory, UMR 5234 CNRS-University of Bordeaux, 33076 Bordeaux, France; 3 Viral DNA Integration and Chromatin Dynamics Network (DyNAVir), 33076 Bordeaux, France

**Keywords:** retrovirus, foamy virus, integrase, integration

## Abstract

Foamy viruses (FV) are retroviruses belonging to the *Spumaretrovirinae* subfamily. They are non-pathogenic viruses endemic in several mammalian hosts like non-human primates, felines, bovines, and equines. Retroviral DNA integration is a mandatory step and constitutes a prime target for antiretroviral therapy. This activity, conserved among retroviruses and long terminal repeat (LTR) retrotransposons, involves a viral nucleoprotein complex called intasome. In the last decade, a plethora of structural insights on retroviral DNA integration arose from the study of FV. Here, we review the biochemistry and the structural features of the FV integration apparatus and will also discuss the mechanism of action of strand transfer inhibitors.

## 1. Introduction

The *retroviridae* family is a large group of viruses containing seven genera (alpha, beta, gamma, delta, epsilon lenti, and spuma-virus). The deltaretrovirus and lentivirus genera contain the two major human pathogens, Human T-Lymphotropic Virus (HTLV-1) and Human Immunodeficiency Virus-1 (HIV-1), respectively. One feature that distinguishes retroviruses from the other viruses is the ability to integrate their linear double stranded DNA into host cellular chromatin. This essential activity is catalyzed by the virally encoded integrase (IN) protein and will lead to the covalent insertion of the provirus into the host genome [1]. The mechanism of retroviral integration is also shared by numerous prokaryotic and eukaryotic mobile DNA elements to mobilize genetic information between and within genomes. Moreover, retroviral integrases are closely related to the DD(E/D) polynucleotidyl transferase family of DNA transposases [2]. Although the DNA cutting and strand transfer reactions occur through a similar mechanism between these genetics elements, the structure of DNA to be mobilized differs, i.e., IN cannot act on an already integrated DNA molecule and requires linear DNA to carry out the two essential sequential events, 3′ processing, and strand transfer [3,4,5]. These processes take place in the context of a nucleoprotein complex called intasome, consisting of the two viral DNA (vDNA) ends and a multimer of IN [6,7]. While the function of retroviral integrases is well described, the molecular mechanisms involved were, for a long time, hampered by the lack of structural information. The propensity of many retroviral integrase to self-associate into high order aggregates in vitro has been a factor limiting structural endeavors. Conversely, FV integrase like prototype foamy virus (PFV) was shown to be very amenable for structural biochemistry and was the source of many breakthroughs on the comprehension on the molecular basis of retroviral integration and strand transfer inhibitors resistance [8,9,10,11]. 

## 2. Biochemistry of Foamy Virus Integration

**Figure 1:**
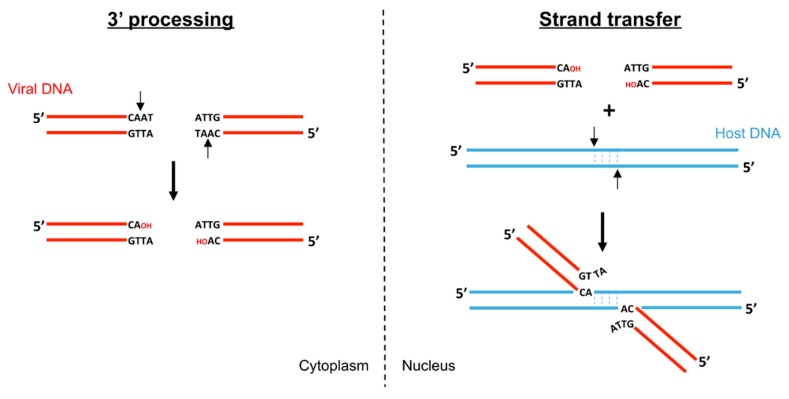
DNA cutting and joining steps catalyzed by retroviral integrases. During 3′ processing (**left**) the integrase removes two (or three) nucleotides from the 3′ ends to expose a conserved terminal CA dinucleotide. The 3′ hydroxyl groups (red OH) will be used in the second step (**right**) to attack the phosphodiester bonds on each target DNA strand.

Biochemical studies of retroviral integration started with the purification of preintegration complexes (PIC) from infected cells [12,13]. Such complexes can perform vDNA integration into target DNA in vitro. Analysis of the intermediates produced during these integration reactions uncovered the two activities catalyzed by retroviral integrase: 3′ processing and strand transfer (Figure 1) [3,4]. The resulting integration products generate a single strand gap and a two-nucleotide overhang that will be repaired by cellular proteins to complete the integration reaction.

During 3′ processing, retroviral integrase cleaves two (or, depending on the in vitro conditions, three [14,15]) nucleotides on the 3′ ends of the U3 and U5 vDNA long terminal repeats (LTR). This sequence-specific reaction, a nucleophilic attack by a water molecule, liberates a recessed 3′ hydroxyl group adjacent to an invariant CA dinucleotide [5]. Foamy virus 3′ processing occurs asymmetrically, modifying only the U5 end as the U3 extremity generated after reverse transcription constitutes a bona fide substrate for integration [16,17]. In contrast, the U5 extreme dinucleotides are necessary during the first strand of reverse transcription but have to be cleaved off for integration. During the strand transfer step, the intasome binds host chromosomal DNA, forming the target capture complex (TCC), and utilizes the 3′ hydroxyls as nucleophiles to cut and join simultaneously both 3′vDNA ends to apposing DNA strands with 4–6 bp stagger (4 in the case of FV).

Recombinant retroviral integrases are very efficient at catalyzing 3′ processing and strand transfer reactions in vitro [18,19,20]. However, the bulk of strand transfer products obtained are generally the result of unpaired products, also called half site integration. Recombinant PFV integrase became a standard model to investigate retroviral integration, as it appeared far more proficient at paired full-site integration. PFV integrase is more soluble in vitro than HIV-1 IN, but the exact biochemical reasons underlying these differences are unclear. Interestingly, comparison of in vitro IN enzymatic reaction conditions among FVs, such as substrate specificity, cofactor usage, and target commitment, showed that the feline foamy virus (FFV) IN has a broader range of substrates and cofactor than other FV INs [21]. FFV IN cleaved PFV U5 LTR substrate, as well as FFV U5 LTR substrate, during in vitro 3′ processing reaction, but not vice versa. The internal six nucleotides in front of terminal CA dinucleotide are identical between the two substrates, indicating that the FFV IN has low substrate specificity compared with PFV IN. Mn^2+^ or Mg^2+^ ions are known as essential cofactors of IN enzyme activities, and in vitro IN activities appear most effectively in the presence of Mn^2+^. Previous studies have reported that multimerization of HIV-1 IN was promoted by Ca^2+^ as well as Mn^2+^, although Ca^2+^ could not substitute in strand transfer reaction [22]. Interestingly, Zn^2+^ and Ca^2+^ divalent cations were found to act in FFV 3′ processing in the absence of Mn^2+^ ion, and their inductions of enzymatic reactions were concentration-dependent. Moreover, like FFV IN, PFV integrase was shown to be fairly lax for divalent cations and target DNA commitment. Indeed, while HIV-1 integrase was shown to commit to substrate DNA within 1 min, PFV integrase took more than an hour [23]. Moreover, the same group performed single molecule experiments using PFV intasomes to investigate the mechanics of target DNA capture and catalysis. Using single molecule total internal reflection fluorescence (smTIRF) microscopy, individual PFV intasome were visualized on naked DNA [24]. Theoretical dynamic modelling showed a 1D rotation-coupled translational diffusion of PFV intasome along DNA. 1D diffusion is a phenomenon exploited by many proteins to scan for sequences, lesions, or structures on nucleic acids. Remarkably, this target DNA searching process is very often non-productive as few integration events were recorded, even in the presence of favored PFV integrase sequences. Instead, since PFV intasome prefers supercoiled DNA as the target substrate [8,24], the authors suggested an additional search for DNA conformation rather than sequence alone. However, the question of the search process on the nucleosomal chromatin template remains to be investigated.

## 3. Domain Organization of Retroviral Integrase

**Figure 2:**
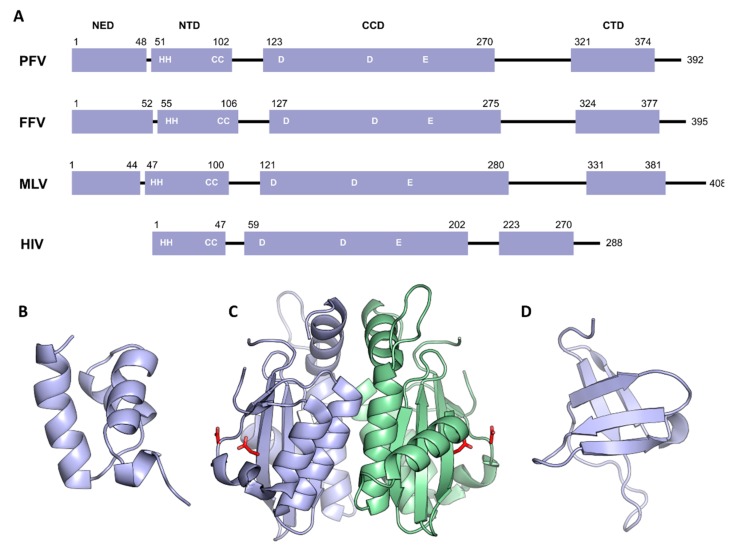
Domain organization of retroviral integrases. (**A**) Schematic of the retroviral IN domain sequences shown as boxes. Isolated domain structures of HIV-1 NTD (**B**), (PDB 1WJC), CCD (**C**), (PDB 1ITG), CTD (**D**), (PDB 1IHV). Chains are shown in cartoon, except active site residues Asp64 and Asp116, which are shown as red sticks (Glu152 residues are disordered and not visible in the structure).

All retroviral IN contain three conserved folded domains that were initially identified using limited proteolysis on HIV-1 IN [25]: the N-terminal domain (NTD), the catalytic core domain (CCD), and the C-terminal domain (CTD). In addition, *spumaretrovirinae* (as well as epsilon and gammaretroviral) integrases harbor a ~40 residues NTD extension domain (NED) (Figure 2A). 

The first structural features of individual domains were obtained using nuclear magnetic resonance (NMR) and X-ray crystallography. The structure of HIV-1 and HIV-2 NTD was determined using NMR and shows 3-helical bundles coordinating a single zinc atom via the side chains of a HisHisCysCys (HHCC) motif [26,27] (Figure 2B). The structure confirms the importance of the zinc as an IN cofactor, and also the location of the conserved His and Cys residues involved in the chelation of metal. The CTD structure was also solved in solution by NMR and revealed a high similarity with Src homology 3 (SH3)-like beta barrel and Tudor domains [28,29] (Figure 2D). The NTD and CTD domains play important roles in substrate recognition and assembly of intasome. They are connected to the CCD via flexible linkers whose size varies among retroviral genera. The CCD contains the active site of the enzyme with the invariant D,D-35-E motif. The crystal structure of HIV-1 IN CCD showed a nucleotidyltransferase fold, which is shared with several prokaryotic and eukaryotic transposases, recombinases, and resolvases [30,31]. The structure revealed a dimer of CCD with an extensive interface. The two active sites are facing outward, opposite to each other, and separated by approximately 35 Å. This distance is incompatible with a functional concerted integration of the two viral ends across a major groove of the target DNA that is around 17 Å in a canonical B-form (Figure 2C). Following this observation and the similarity with the mechanistically related transposases [32,33,34], it appeared clear that an IN multimer must be involved in vDNA concerted integration. Biochemical analysis of IN from various genera failed to establish a relationship between their oligomeric states in solution and the formation of active complexes once bound to their cognate DNA substrates. The breakthrough came from PFV integrase. Monomeric in solution, highly soluble, and exceptionally efficient in catalysis in vitro, this model was the first functional retroviral IN.DNA complex amenable to structural characterization.

## 4. Architecture of the PFV Intasome

Determined by X-ray crystallography, the structure of the PFV intasome fundamentally changed the landscape in the field of retroviral integration, as it could both unravel the functional architecture of the integration apparatus and elucidate the mechanism of action of HIV strand transfer inhibitors [9].

**Figure 3:**
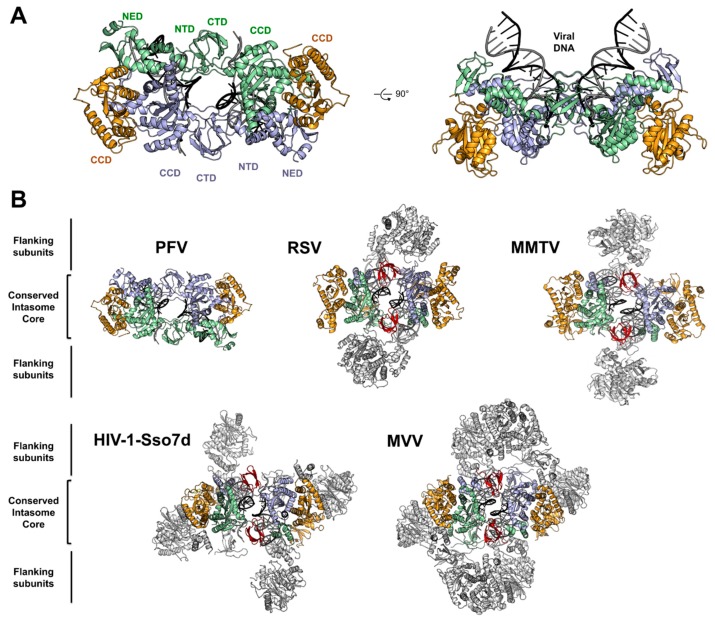
Architecture of PFV and related retroviral intasomes. (**A**) PFV intasome shown in two orthogonal views (PDB 4E7H) with individual domains indicated. Inner IN subunits are colored pale green and light blue, and the outer subunits are in orange. (**B**) Comparison of retroviral intasomes structures (RSV PDB: 5EJK, MMTV PDB: 3JCA, MVV PDB: 5M0Q). Complexes are viewed from below the active site. The conserved intasome core, CIC, is colored as in (A), synaptic CTDs are in red, and flanking subunits are in light grey.

The PFV intasome revealed a tetramer of integrases synapsing a pair of vDNA ends. The tetramer consists of a dimer of dimer with two structurally distinct subunits (Figure 3A). The inner subunits mediate all the protein–protein, protein–DNA contacts in an extended conformation and host the active sites to catalyze the 3′ processing and strand transfer reactions. The inner integrases interact via intermolecular NTD−CCD contacts, and by the insertion of a pair of CTDs that rigidly bridge the two halves of the intasome between the CCDs. The outer subunits connect the inner protomers via the canonical CCD–CCD interface. Although the respective positions of the outer NTDs and CTDs are not resolved in the intasome structures published to date, some hints were obtained using SAXS/SANS analysis of PFV intasome [35]. These domains are dispensable for PFV intasome assembly and in vitro activity [36] but they are suspected to provide additional stabilizing interaction with vDNA and/or cellular cofactors. However, the outer CTDs appear to promote aggregation in vitro, as further experiments using intasome lacking the outer domains have shown an increased stability and activity on naked DNA. Solving the structure of the PFV intasome reinforced the hypothesis that the tetrameric architecture was the functional multimer of HIV-1 intasome. Yet, more recently, four additional structures from orthoretroviral intasome; α-retroviral Rous sarcoma virus (RSV) [37], β-retroviral mouse mammary tumor virus (MMTV) [38], lentiviral maedi-visna virus (MVV) [39], and lentiviral HIV-1 [40] were reported, revealing a variety of architectures (see [41] for a more detailed review) (Figure 3B). First, RSV and MMTV intasomes structures solved by X-ray crystallography and Cryo-EM, respectively, revealed an octameric assembly. A core tetramer (called conserved intasome core, CIC [41]) is positioned similarly as in PFV intasome, with the conserved inner catalytic protomers flanked by outer monomer subunits. The position of the synaptic CTDs bridging both halves of the intasome is conserved in the octameric structures, but due to the small size of the CCD–CTD linker, they cannot be supplied by the inner protomer and come from the flanking dimers. Indeed, while in PFV IN the CCD–CTD linker is fifty residues long, in α and β retroviral INs, they are only eight amino acids long. Interestingly, the size of this linker varies among retroviral genera and may predict the requirement for additional oligomers to support CIC assembly [38]. 

In the case of lentiviral (and δ-retroviral) INs, the size of the CCD–CTD linker is around twenty residues. However, it adopts a compact alpha-helical structure, which is predicted to be incompatible to allow the formation of a minimalist CIC [42]. 

Fusing HIV-1 IN with the DNA binding domain Sso7d [43] promoted its solubility as well as its in vitro activity [44], allowing the assembly of a complex that could be structurally characterized by Cryo-EM. The structure of the HIV-1-Sso7d intasome revealed a tetramer competent for integration [40]. However, the CCD–CTD linker could not be seen on the electron density map, and assembly of an intasome using HIV-1 IN cofactor lens epithelium-derived growth factor (LEDGF/p75) integrase binding domain (IBD) to stabilize higher-order species revealed a dodecameric structure. In this complex, the core intasome is assembled between two tetramers with a flanking dimer inserting the synaptic CTDs.

The MVV intasome was assembled using wild type integrase proteins and shows a hexadecameric structure (a tetramer of tetramers). Here again, the catalytic core is formed by the CIC. Overall, both intasome architecture are similar and resume the CIC formation. It has been suggested that the extra fusion domain Sso7d in HIV-1 intasome, which cannot be seen in the EM density, may disrupt the dimer–dimer interaction in the flanking HIV-1 IN tetramer, and therefore result in a dodecameric structure, while MVV intasome displays a hexadecamer.

## 5. Structural Basis for Target DNA Capture

**Figure 4:**
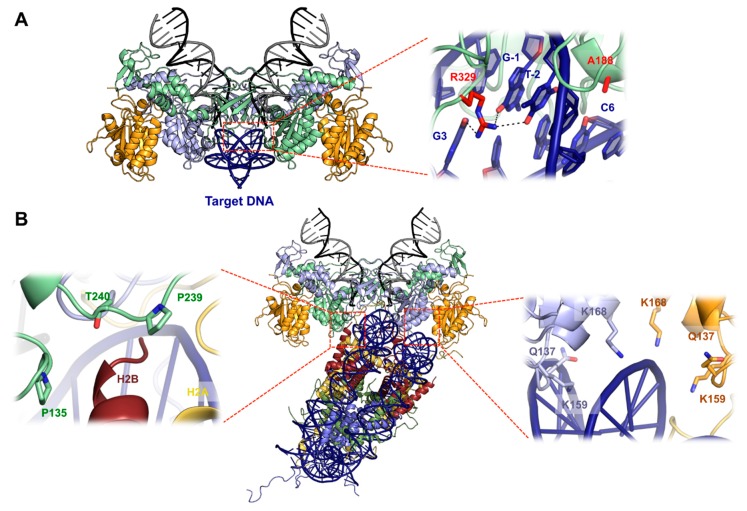
Target DNA capture. (**A**) Crystal structure of the target capture complex TCC (PDB: 3OS1) with sequence specific target DNA interactions shown as a blow up. Arg329 making contacts with guanosine 3, −1, and thymine −2, as well as Ala188 making contact with cytosine 6, are shown as red sticks. (**B**) Structure of the PFV intasome–nucleosome complex displayed as pseudoatomic model by docking PFV intasome (PDB 3L2Q) and nucleosome (PDB 1KX5) structures into the Cryo-EM map (EMDB ID 2992). Histones H2A are colored in yellow, H2B in red, H3 in blue, and H4 in green. IN contacts with H2B (**left**) and with the second gyre of nucleosomal DNA (**right**) are shown as zoomed boxes.

Co-crystallization of the PFV intasome with its target DNA (tDNA) allowed the visualization of both target capture complex (TCC) and strand transfer complex (STC) before and after the reaction, respectively [10,45]. The tDNA binds along the groove created by the two inner subunits, right below the active site (Figure 4A). The intasome does not undergo significant structural rearrangements to accommodate the tDNA, which is severely bent. This deformation is maximal at the center of the integration site, with the widening of the major groove to 26.3 Å. This separation allows the scissile phosphodiester to fit into the active site for in line nucleophilic attack. Because DNA bendability is in large part dictated by the nature of the dinucleotide step, with pyrimidine–purine (YR) being the most flexible and purine-pyrimidine (RY) being the least, it is then not surprising that PFV integration sites are naturally biased towards more flexible pyrimidine–purine dinucleotide at the central position. As expected, due to the low selectivity of tDNA sequence, the majority of contacts between the intasome and tDNA are mediated through the phosphodiester backbone [10], except CCD residue Ala188 and CTD Arg329, that make base-specific contacts. Ala188 makes van der Waals interaction with cytosine at position 6, whereas Arg329 interacts with guanosine 3, guanosine −1, and thymine −2 through hydrogen bonds (Figure 4A, right). Interestingly, these two residues interact with all the consensus bases flanking the flexible central YR dinucleotide. Consequently, PFV IN Ala188 and Arg329 mutants showed in vitro strand transfer defects, as well as new sequence selectivity. The importance of these contacts has been validated for HIV-1 integrase, as mutating Ser119 (the structural equivalent of PFV IN Ala188) showed altered strand transfer and modified sequence selectivity [46,47,48]. 

In eukaryotes, host target DNA is compacted within chromatin that strongly distorts DNA around nucleosomes. PFV intasome showed strong integration activity when supplied with purified or recombinant human mononucleosomes [11,49]. Isolation of a stable complex of the PFV intasome and recombinant mononucleosome permitted the characterization by cryo-electron microscopy (Cryo-EM) of the TCC and a nucleosome core particle at 8 Å resolution [11] (Figure 4B). The crystal structures of the intasome and the nucleosome can be unambiguously docked into the electron density map. The intasome harbors the classical tetramer with the two types of subunits. No additional density is seen compared to the previous intasome crystal structures. The intasome sits on nucleosomal DNA above one of the H2A–H2B dimers and makes an extensive nucleosome–intasome interface involving three IN subunits, both turns of the nucleosomal DNA, and one H2A–H2B dimer. The carboxy-terminal helix of H2B is directly poking toward the intasome and is surrounded by a triad of loops from the inner subunits. Integrase residues Pro135, Pro239, and Thr240 wrap the C-terminal helix of H2B (Figure 4B, left) and the double substitution P135E/T240E strongly affected nucleosome binding and nucleosome strand transfer activity. The histone H2A shows density from its N-terminus reaching out to the inner IN CTD, and deletion of the first twelve H2A residues abolished intasome binding and decreased strand transfer activity into nucleosome. Further mutagenesis uncovered a role for the intasome outer domains, specifically the outer CTDs, as its deletion reduced the ability to bind nucleosomes. Additional important contacts between the intasome and the nucleosome involve the canonical CCD–CCD interface and the second gyre of nucleosomal DNA (Figure 4B, right). Residues Q137, K159, and K168 are located in the vicinity of the contacts with the second gyre of DNA, and their substitution affected nucleosome binding and integration activity in vitro.

Most striking is the path of DNA captured within the tDNA-binding groove of the intasome. When compared to its structure on a native nucleosome, the captured DNA is kinked and lifted from the surface of the histones, perfectly matching the strong bending seen on the PFV intasome capture complex [11]. The multivalent intasome–nucleosome interactions may aid to reach the energy state required to deform nucleosomal DNA beyond its ground state, and seems to be the only determinant required as, more recently, Yoder and colleagues demonstrated that unwrapping DNA-histones modifications in the vicinity of the intasome integration sites does not impact nucleosome capture [50].

## 6. Mechanics of PFV Intasome Active Site

**Figure 5:**
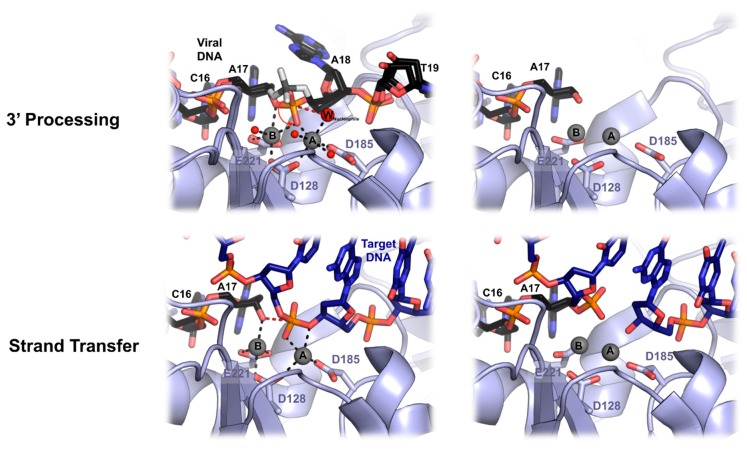
Mechanics of PFV 3′processing and strand transfer. Top panel, a close up of PFV intasome active site during 3′ processing. Superimposition of intasome structures before 3′ processing with and without bound manganese Mn^2+^ (grey spheres A and B) (**left**) and after cleavage (**right**). Relocation of the scissile phosphodiester upon metal binding is shown with a red arrow. The red spheres illustrate the water molecules, and the nucleophile water molecule is shown as a big red sphere. Bottom panel, strand transfer activity upon target DNA binding. The nucleophilic attack is shown with red dashes.

Because the IN catalysis requires divalent metal ion cofactor, it has been possible to freeze the PFV enzyme in different ground states before 3′ processing and strand transfer [45] (Figure 5). Both reactive and non-reactive strands of the vDNA are separated via the intrusion of the residues Pro214-Gly218, stacking against the adenine base, leaving three bases unpaired. The scissile dinucleotide phosphodiester backbone makes hydrogen bonds with Tyr212 and Gln186, while the adenine and thymidine bases contacts with the IN are limited to Van der Waals interactions. The binding of the two Mn^2+^ ions in the active site induces a shift of the scissile phosphodiester toward the catalytic triad DDE. The metal ion A is in a near perfect octahedral coordination. It comprises oxygen atoms from Asp128 and Asp185, the pro-S_p_ oxygen atom of the scissile phosphodiester and three water molecules, one of them positioned for in-line nucleophilic attack on the scissile CA\AT phosphodiester bond. Both oxygen atoms of Glu221 and one from Asp128 coordinate metal B, as well as one water molecule, a bridging oxygen atom of the scissile phosphodiester and a non-bridging pro-S_p_ oxygen shared with metal A. This non-ideal environment for metal B may aid scissile phosphodiester bond destabilization during catalysis. Before 3′ processing, the distance between the two metal ions is 3.9 Å, and changes to 3.1 Å after dissociation of the dinucleotide. This metal ions movement has been also described in the RNase H active site and was suggested to allow the nucleophilic water to approach the scissile phosphodiester [51]. In the active site, the metal cofactors move further apart from each other (from 3.1 Å to 3.8 Å) upon target DNA capture. The roles of both metal ions changes between 3′ processing and strand transfer. Metal A and metal B coordination with active site residues stays unchanged, as well as the sharing of the pro-S_p_ oxygen atom from the target phosphodiester. Accordingly, metal A destabilized the target phosphodiester scissile bond by interacting with the 3′-bridging oxygen atom while metal B activates and positions the 3′OH of the vDNA for nucleophilic attack. After strand transfer catalysis, both metal ions move closer to approximately 3.2 Å.

Overlaying the TCC and the STC structure shows that the overall DNA conformations do not change, except the position of the phosphodiester linking the tDNA to vDNA, which is shifted away from the active site. Integrase apply a significant torsional stress to the tDNA, likely providing the displacement force, which is relieved upon cutting of the target phosphodiester bond. This ejection prevents any reversible reaction that would lead to unfruitful viral infection. A soaking experiment with metal cofactor showed an apparent loss of metal B binding affinity after strand transfer, probably due to the ejection of the DNA from the active site. Interestingly, such a tDNA kink within the active site is important for other transpososomes activity like Hermes [34,52], MuA [53], Tn10 [54], and IS231A [55]. This could be an evolutionary conserved feature of DNA transposition apparatus in order to prevent any reversal reaction, while being competent to access tDNA scissile phosphodiester.

## 7. PFV Intasome and HIV-1 Strand Transfer Inhibitors

**Figure 6:**
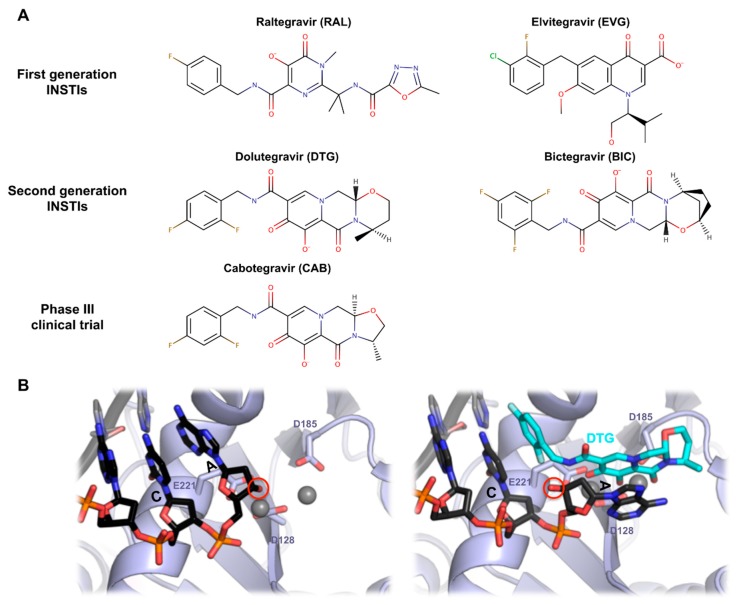
Integrase strand transfer inhibitors. (**A**) Chemical structures of INSTIs. (**B**) Structure of PFV active site with or without DTG. By positioning into the active site, the INSTI engages the metal cofactors and induces a shift of the 3′ reactive hydroxyl (red circle) out of position incompatible for strand transfer.

Human immunodeficiency virus type 1 (HIV-1) IN has been widely considered as an important target protein for novel anti-acquired immune deficiency syndrome (AIDS) drugs [56]. Based on biochemical assay and biophysical analysis, several classes of retroviral IN inhibitors have been discovered over the last 25 years [57,58,59,60]. Hydroxylated natural products and their derivatives were developed, and the most important IN inhibitor family, diketo acids (DKA), emerged [59]. Integrase strand transfer inhibitors (INSTIs) are one of active site inhibitors against HIV-1 integration that act by preventing the strand transfer reaction; however, numerous significant developments and rational designs of INSTIs were reported during recent years. Raltegravir (RAL) was the first INSTIs approved by the United State food and drug administration (FDA) in 2007 [61], providing a new option for highly active antiretroviral therapy (HAART). After that, elvitegravir (EVG) and dolutegravir (DTG) have been approved [62,63] (Figure 6A). RAL, EVG, and DTG belong to the bioisosteres compounds of DKA. DKA derivatives, which contain a 1,3-dicarbonyl aromatic ring, are a class of highly effective HIV-1 INSTIs where the 1,3-dicarbonyl group seizes two Mg2+ ions, preventing the metal ion-mediated retroviral integration [64,65,66]. More recently, two new molecules, bictegravir (BIC) and cabotegravir (CAB), have been developed [67,68]. Bictegravir was approved by the FDA in early 2018 and is being used as a combination drug. Cabotegravir is currently in phase III development. BIC and CAB are structurally similar to DTG with their tri-cyclic central pharmacophores (Figure 6A), but the latter offers an improved half-life [69].

Despite an increasing drug arsenal, the experimental data related to full-length, wild type HIV-1 intasomes structures are rare. As an alternative, PFV intasome has been adopted for anti-AIDS drug development. A comparison of the CCD structures between HIV-1 and PFV showed that both conserved unique structural features, such as the host cellular factor binding faces and the organization of the active site [8,9,30]. A recent NMR study using the CCD of HIV-1 IN showed that the HIV-1 and PFV IN flexible loops (residues 140–149 in HIV and 209–218 in PFV) are almost similar, and structure prediction of the HIV integrase intasome provided further evidence for the similarities between the active amino acid resides of the PFV and HIV INs [70,71]. Johnson et al. generated a corresponding HIV-1 IN model from the PFV IN crystal structure and they predicted the in vitro anti-INSTI activities using molecular docking and molecular dynamics simulation [72]. Despite the limited sequence similarity and different intasome architecture features (lentiviral: tetramer-of-tetramer, PFV: dimer-of-dimer), PFV IN was highly sensitive to HIV INSTIs [8,73], suggesting that INSTIs target the most conserved regions of IN-DNA complexes. Recently, many studies using PFV IN as a surrogate model in order to investigate HIV-1 INSTIs have been published. Some groups investigated the consistency between in vitro and in vivo resistance profiles for RAL using PFV IN structures mutated at the corresponding to HIV-1 IN active site, Q148, and N155 [74,75]. Hare et al. confirmed the interaction of the pharmacophores of PFV intasome with two metal ions at the IN active site, and they also investigated an interaction between the bound vDNA end and the benzyl group through cocrystal structures of RAL and EVG [9]. Also, they obtained a crystal structure of PFV IN complexed with vDNA and DTG [76] (Figure 6B) (See [1] for more details on the structural basis for INSTIs). Johnson et al. designed a series of INSTIs based on the previous target model through homology modeling and structural superposition method. In their modeling system, the junction between the CCD and CTD adopts a helix-loop-helix motif, which is similar to the corresponding segment of PFV IN [77].

Hu et al. investigated the inhibitory mechanism of RAL and the recognition of DKA inhibitors with PFV-IN via molecular dynamics and molecular docking methods, and they validated the HIV-1 inhibitor screening platform [73,78]. Du et al. proposed the crystal structure of PFV-IN DNA as a potential HIV-1 INSTI screening platform through a structural biology information survey [79]. They also investigated the molecular recognition system of PFV IN, using six naphthyridine derivatives inhibitors through molecular docking, molecular dynamics simulations, and water-mediated interactions analyses. Besides, there are a lot of studies using PFV intasome to explore the binding mode of compounds for new HIV IN inhibitors. These results have implications for the rational design of HIV-1 IN targeting specific INSTIs with improved affinity and selectivity [80,81]. 

Some studies have raised doubts on HIV-1 IN inhibitor screening platforms using PFV-IN, indicating that the HIV-1 IN system behaves differently from PFV in terms of folding, recognition, and hydrophobicity of the tDNA binding site, and stability [82]. Although conformational changes and the energy landscape are still unclear, the molecular docking and molecular dynamics study validates the reliability of the platform and reestablishes PFV IN as one of the most credible surrogate model for HIV-1 INSTIs studies and anti-AIDS drug development based on IN structure. Nevertheless, thanks to Cryo-EM advances, future high-resolution structures of primate lentiviral integrases will be of great interest to further improve the structural basis of INSTI mechanisms and development.

## 8. Conclusions and Perspectives

As an important therapeutic target and molecular tool, retroviral integrase is having a lot of attention from the scientific community. Intensive biochemical studies gave important insights on the functional architecture of the viral enzyme and, little by little, the structural counterpart emerged: from individual domains to active intasomes bound to a nucleosome. The publication in 2010 of the first retroviral intasome structure from PFV was the starting point of a decade-long period of exciting and insightful research on the integration process. The recent revolution in single particle cryo-electron microscopy significantly increased the repertoire of retroviral intasome structures now available that highlight both the conservation and diversity in the architectures. Conservation, because the presence on all retroviral intasome of a PFV-like intasome CIC hosting the catalytic subunits is quite striking, and diversity being on the variety of oligomers needed for the whole assembly. It will be of great interest to expand the catalogue of known intasome structures from the remaining retroviral genera, but also to further investigate new structures derived from wild type primate lentiviral integrases to better understand HIV-1 strand transfer inhibitors. 

Many open questions will surely keep the fire of retroviral integration research vivid, notably, what is the precise chronology of intasome assembly during infection. Indeed, HIV-1 virion packages around 250 molecules of integrase, which is far more than needed from the recent structures of lentiviral intasomes. Also, although the structure of the PFV intasome bound to a nucleosome afforded important information on the chromatin capture by retroviral intasomes, the requirement for histones might differs from genus to genus [83,84], highlighting the need for additional structures of intasomes bound to nucleosomes. Additionally, early chromatinisation of retroviral pre-integration complexes has emerged as a feature of two retroviral genera [85,86]. Future studies will be required to determine the functional importance and the conservation among integrative mobile elements and, notably, Foamy viruses.
